# Cross‐sectional serum metabolomic study of multiple forms of muscular dystrophy

**DOI:** 10.1111/jcmm.13543

**Published:** 2018-02-14

**Authors:** Pietro Spitali, Kristina Hettne, Roula Tsonaka, Ekrem Sabir, Alexandre Seyer, Jesse B.A. Hemerik, Jelle J. Goeman, Esther Picillo, Manuela Ergoli, Luisa Politano, Annemieke Aartsma‐Rus

**Affiliations:** ^1^ Department of Human Genetics Leiden University Medical Center Leiden The Netherlands; ^2^ Department of Medical Statistics and Bioinformatics Leiden University Medical Center Leiden The Netherlands; ^3^ Profilomic SA Boulogne‐Billancourt France; ^4^ Cardiomyology and Medical Genetics Department of Experimental Medicine University of Campania “Luigi Vanvitelli” Naples Italy

**Keywords:** biomarkers, Duchenne muscular dystrophy, metabolomics, muscular dystrophy

## Abstract

Muscular dystrophies are characterized by a progressive loss of muscle tissue and/or muscle function. While metabolic alterations have been described in patients’‐derived muscle biopsies, non‐invasive readouts able to describe these alterations are needed in order to objectively monitor muscle condition and response to treatment targeting metabolic abnormalities. We used a metabolomic approach to study metabolites concentration in serum of patients affected by multiple forms of muscular dystrophy such as Duchenne and Becker muscular dystrophies, limb‐girdle muscular dystrophies type 2A and 2B, myotonic dystrophy type 1 and facioscapulohumeral muscular dystrophy. We show that 15 metabolites involved in energy production, amino acid metabolism, testosterone metabolism and response to treatment with glucocorticoids were differentially expressed between healthy controls and Duchenne patients. Five metabolites were also able to discriminate other forms of muscular dystrophy. In particular, creatinine and the creatine/creatinine ratio were significantly associated with Duchenne patients performance as assessed by the 6‐minute walk test and north star ambulatory assessment. The obtained results provide evidence that metabolomics analysis of serum samples can provide useful information regarding muscle condition and response to treatment, such as to glucocorticoids treatment.

## 1 INTRODUCTION

Inherited muscular dystrophies (MDs) are caused by mutations in multiple genes.^1^ The disease is characterized by progressive loss of muscle tissue and muscle function. Duchenne muscular dystrophy (DMD) is the most common and most severe MD. It is a lethal disease caused by lack of dystrophin due to protein‐truncating mutations in the *DMD* gene.^2^, ^3^, ^4^, ^5^ The identification of non‐invasive biomarkers able to monitor disease progression and response to therapy would enable better patients’ management and faster evaluation and marketing authorization of medicinal products for DMD and MDs in general. Towards this aim, multiple groups are working to identify biomarkers in body fluids such as blood‐derived samples and urine.^6^, ^7^, ^8^, ^9^, ^10^, ^11^, ^12^, ^13^, ^14^, ^15^ Most of the available information was obtained by studying protein concentrations in different samples matrices and miRNAs, while less information is available for metabolites concentration in body fluids, even though DMD was considered in the past and recently re‐evaluated to be a metabolic myopathy.^16^ Recently markers of metabolic syndrome such as serum levels of leptin,^17^ creatine, arginine, branched amino acids and phosphatidylcholine^18^, ^19^, ^20^ were reported to be elevated in DMD, leading us to further study metabolites profiles in DMD patients.

The aims of this study were (i) to identify metabolites able to discriminate between patients and controls, and (ii) to test associations between biomarkers levels and clinical performance as measured by the 6‐minute walk test (6MWT) or the North Start Ambulatory Assessment (NSAA).

Furthermore, we investigated whether the observed signature could be translated to other MDs, such as the milder allelic variant Becker muscular dystrophy, but also genetically separate myotonic dystrophy type 1 (CUG expansion in the *DMPK* gene), facioscapulohumeral muscular dystrophy (shortening of a subtelomeric repeat unit enabling the production of the toxic *Dux4* retrogene) and limb‐girdle muscular dystrophies type 2A and 2B (lack of calpain‐3 and dysferlin, respectively).

## 2 MATERIALS AND METHODS

### 2.1 Participants

Patient and healthy control samples were obtained from the Naples Human Mutation Gene Biobank (NHMGB), partner of EuroBioBank and of the Telethon Network of Genetic Biobanks. A total of 30 DMD (mean age 9.3 years), 10 Becker muscular dystrophy (BMD, mean age 10.8 years), 10 facioscapulohumeral muscular dystrophy (FSHD, mean age 39.6 years), 10 myotonic dystrophy type 1 (DM1, mean age 43.9 years), 5 limb‐girdle muscular dystrophy type 2A (LGMD2A, mean age 32.6 years), 5 LGMD2B (mean age 29.2 years), 10 paediatric controls (mean age 15.4 years) and 12 adult controls (mean age 46.4 years) were included in the study. Blood was drawn in the morning, and all individuals had fasted since the previous day. Metadata connected to this cohort are shown in Table S1.

The study was approved by the Institutional Review Board. Informed consent forms for blood drawing were obtained for all participants, at the time of the scheduled follow‐up. The investigation was conducted according to the declaration of Helsinki.

### 2.2 Serum preparation

Venous blood was allowed to clot for 30 minutes in red‐capped tubes followed by centrifugation for 10 minutes at 2350 *g*. Serum was carefully removed, aliquoted and stored at −80°C pending use.

### 2.3 Data acquisition

Experiments were performed as previously detailed.^21^ Briefly, 15 μL of serum samples was used for protein precipitation using methanol containing a mixture of internal standards (Table S2). Samples were then mixed, centrifuged, dried under nitrogen and resuspended in 45 μL of 10 mmol/L of ammonium carbonate (pH 10.5)/ACN, 40/60 (v/v). Serum samples were then introduced into a Transcend 1250 LC system (Thermo Fisher Scientific, Les Ulis, France) fitted with a Sequant ZICpHILIC 5 μm, 2.1 × 150 mm (Merck, Darmstadt, Germany) and coupled to a Q‐Exactive mass spectrometer (Thermo Scientific, San Jose, CA) operating in both positive and negative ionization modes, alternatively. In positive ion mode, positive ions are detected while in negative ion mode, negative ions are detected. Quality control (QC) samples composed of an equal amount of each sample were discarded all along the analytical sequence. Data analysis was performed with TraceFinder 3.1 (Thermo Fisher Scientific, Les Ulis, France). Compound identification was carried out by comparing their exact *m/z* ratio of their corresponding protonated ion [M+H]^+^ in positive ion mode and of their deprotonated ion [M‐H]^‐^ in negative ion mode, their retention time and their isotopic pattern to an in‐house chemical library. The obtained data set was cleaned based on several parameters: the coefficient of correlation between serial dilutions of QC samples, the coefficients of variation of the areas of chromatographic peaks of features in QC samples and the ratio of chromatographic area of biological to blank samples QC samples.^22^ Data and metabolites identifiers are provided in Tables S3 and S4, respectively.

### 2.4 Statistics and pathway analysis

Data analysis was performed after data standardization. As certain metabolites detected by mass spectrometry in both positive and negative ionization modes, as protonated ions [M+H]^+^ and deprotonated ions [M‐H]^−^, respectively, showed high correlation, we deleted double entries and kept only the ones measured in negative mode. In 4 cases, 2 peaks were assigned to the same chemical ID using the same ionization mode (hexanoylglycine, kynurenic acid, N‐acetyl‐DL‐tryptophan in both positive and negative modes); given the high correlation in the peak area for these instances, we only retained the measurement with higher intensity. To determine differential representation of metabolites in serum, we used a linear model, and the Bonferroni correction for multiple testing was applied. An adjusted *P*‐value of <.05 was considered significant. Linear models were used to investigate potential associations between metabolites levels with age, 6MWD and NSAA. Pathway analysis of the metabolomics data set was performed with the global test.^23^, ^24^ Permutations were used to take into account the correlation between metabolites measurements. Subset's option was used to define pathways obtained from WikiPathways.^25^ Weight's option was used to score each metabolite in order to take multiple mappings of 1 metabolite to a measurement into account. For example, a weight of 1/3 was assigned to measurements with 3 metabolite mappings, while a weight of 1 was assigned to measurements with a single metabolite mapping. The Westfall and Young's maxT method was used to correct for multiple testing. All analyses were performed in R using the *lm, cor, cor.test* and *gt* functions.

### 2.5 Data availability

The data sets generated during and/or analysed during this study are available as supplementary data.

## 3 RESULTS

**Figure 1:**
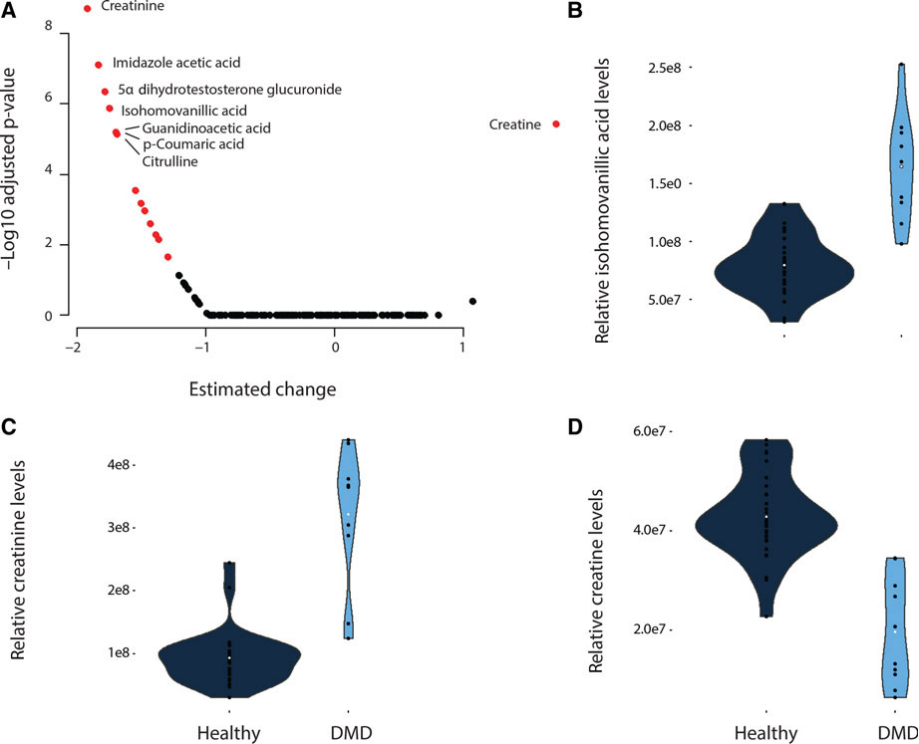
Comparison of metabolites serum levels in DMD and healthy controls. (A) Volcano plot showing the deviation in patients compared to controls (*x* axis) and the −log10 of the adjusted *P*‐value on the *y* axis. Black circles represent metabolites with adjusted *P*‐values below the significance threshold after Bonferroni correction, while red circles represent the 15 metabolites surviving multiple testing correction. (B‐D) Violin plots showing concentration changes for 3 metabolites, namely isohomovanillic acid, creatinine and creatine. Metabolite levels are shown for each patient as black dots, while the white dot represents the mean for each group

Metabolomic analysis of serum samples of 30 DMD patients and 10 healthy age‐matched controls enabled the detection of 227 features in both ionization modes, corresponding to 172 unique compounds. Fifteen metabolites were found to be differentially present in the DMD patients sera compared to age‐matched healthy controls (Figure 1A and Table 1). All metabolites showed reduced levels in DMD patients compared to healthy controls except for creatine (Figure 1B). No or moderate correlations were observed among the 15 metabolites or with creatine kinase activity with the exception of p‐coumaric acid correlating with dehydroisoandrosterone 3‐sulphate (Figure S1). Two metabolites showed a significant positive association with age (Figure S2). While the association between age and dehydroisoandrosterone 3‐sulphate was justified by the data distribution, the association with 5α‐DHT was mostly driven by a few cases.

**Table 1:** List of the 15 metabolites found differentially present in serum of DMD patients compared to healthy control. Fold change, *P*‐values and Bonferroni‐adjusted *P*‐values are reported

Metabolite ID	Fold change	*P*‐value	Bonferroni‐adjusted *P*‐values
Creatinine	0.29	1.10E‐11	1.89E‐09
Imidazole acetic acid	0.30	4.43E‐10	7.61E‐08
5α Dihydrotestosterone glucuronide // androsterone glucuronide // Etiocholan‐3alpha‐ol‐17‐one 3‐glucuronide	0.10	2.59E‐09	4.46E‐07
DL‐p‐Hydroxyphenyllactic acid // Isohomovanillic acid	0.48	7.73E‐09	1.33E‐06
Creatine	2.19	2.10E‐08	3.61E‐06
Guanidinoacetic acid	0.44	3.63E‐08	6.25E‐06
p‐Coumaric acid	0.50	3.76E‐08	6.46E‐06
Citrulline	0.57	4.07E‐08	6.99E‐06
5‐Methoxyindoleacetate // Indoleacetic acid	0.60	1.62E‐06	2.78E‐04
L‐Aspartic acid	0.44	3.93E‐06	6.76E‐04
Ornithine	0.62	6.36E‐06	1.09E‐03
2‐hydroxycaproic acid	0.53	1.46E‐05	2.50E‐03
L‐Serine	0.78	2.91E‐05	5.00E‐03
Dehydroisoandrosterone 3‐sulphate	0.25	4.13E‐05	7.11E‐03
Erythrose	0.46	1.31E‐04	2.25E‐02

**Figure 2:**
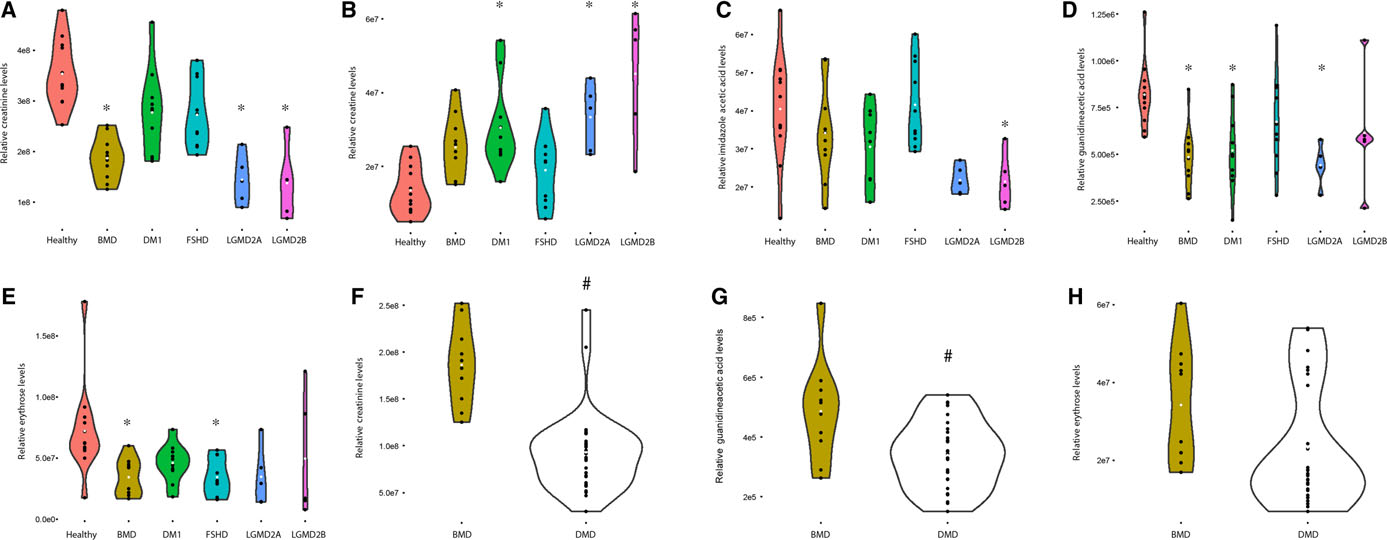
Comparison of metabolites serum levels in other forms of muscular dystrophy. Violin plots are shown to present the distribution of dots for each patients group. Each panel represents data for a different metabolite such as creatinine (panel A), creatine (panel B), imidazole acetic acid (panel C), guanidinoacetic acid (panel D) and Erythrose (panel E). The concentration of metabolites affected in BMD patients was compared to the ones observed in DMD patients (panels F‐H). DMD is Duchenne muscular dystrophy, BMD is Becker muscular dystrophy, FSHD is facioscapulohumeral muscular dystrophy, DM1 is myotonic dystrophy type 1, LGMD2B and LGMD2A are limb‐girdle muscular dystrophies type 2B and 2A. The healthy group is composed by the group of adult healthy people. * indicates significant differences between groups with Bonferroni‐adjusted *P‐*value <.05. # indicates differences between DMD and BMD patients with a *P*‐value <.01

The analysis of the same 15 metabolites in patients affected by other forms of muscular dystrophy such as BMD, DM1, FSHD, LGMD2A and LGMD2B revealed that 5 metabolites were altered in patients compared to controls (Figure 2A‐E). In particular, creatinine levels were significantly lower in BMD (adj. *P *< 10^−5^), LGMD2A (adj. *P *< 10^−5^) and LGMD2B (adj. *P *< 10^−5^); creatine was elevated in DM1 (adj. *P *<* *.01), LGMD2A (adj. *P *<* *.05) and LGMD2B (adj. *P *< 10^−5^). Imidazole acetic acid was decreased in LGMD2B patients (adj. *P *<* *.05); guanidinoacetic acid was decreased in BMD (adj. *P *<* *.05). DM1 (adj. *P *<* *.05) and LGMD2A (adj. *P *<* *.05); erythrose was decreased in BMD (adj. *P *<* *.05) and FSHD patients (adj. *P *<* *.05). Given that creatinine, guanidinoacetic acid and erythrose levels were also affected in BMD patients compared to healthy controls, we compared the concentration of these 3 metabolites between DMD and BMD patients. Creatinine and guanidinoacetic acid were further reduced in DMD compared to BMD (*P *<* *.01), while erythrose levels were comparable between the 2 patients groups (Figure 2F‐H).

**Figure 3:**
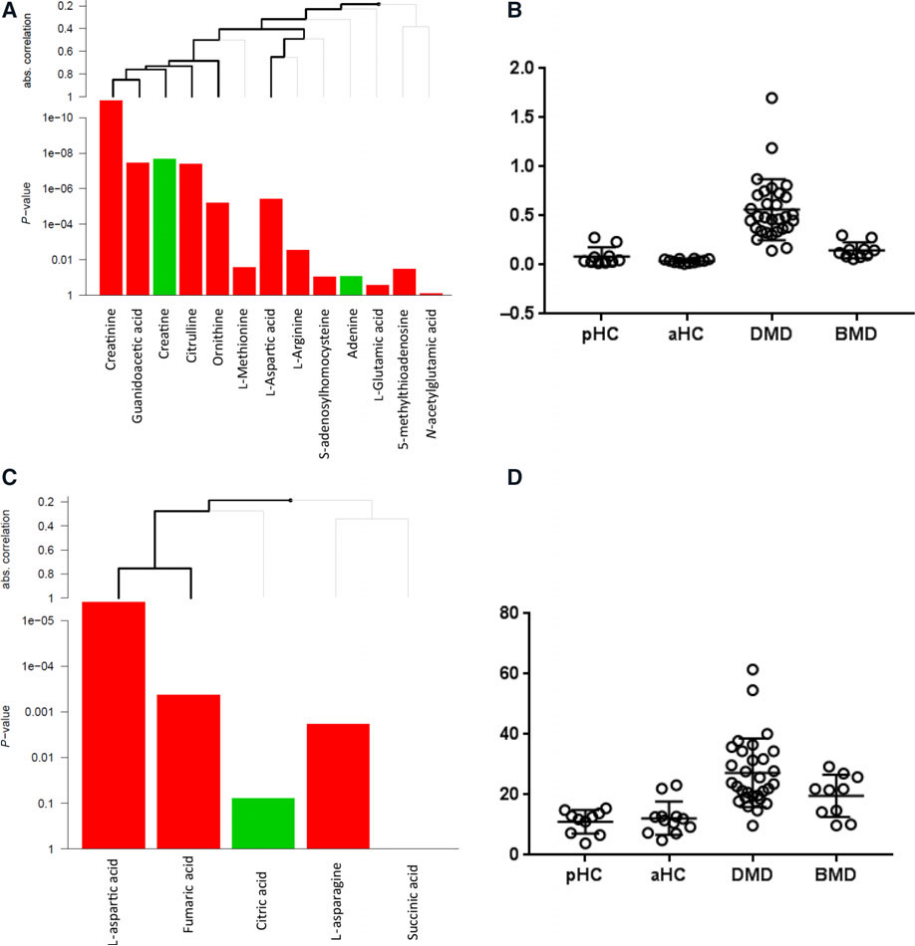
Anticorrelating metabolites mapping to the same pathway. (A) Example of data analysis with the *globaltest* including pathway information. In this example, the contribution of each metabolite to the metabolism of polyamines pathway is presented. Lines above indicate the contribution of each metabolite or of a cluster of metabolites to the pathway score. Thick lines indicate significant contributions. The *P*‐values indicate the contribution of each metabolite to the pathway score. Green bars indicate higher metabolite corresponding area in DMD serum samples, while red bars indicate higher area in healthy control serum samples. (B) Scatter plot showing how the ratio between creatine and creatinine is able to discriminate between DMD, BMD and healthy. pHC is paediatric healthy controls, and aHC is adult healthy controls. (C) Citric acid and L‐aspartic acid opposite contribution to the alanine and aspartate metabolism pathway. (D) Scatter plot showing how the ratio between citric acid and L‐aspartic acid is able to discriminate between DMD, BMD and healthy

To understand and explain the differences between DMD patients and controls, a pathway analysis was performed, which allowed the identification of 23 molecular pathways (Table S5). The metabolism of polyamines was one of the most significant ones with creatine and creatinine anticorrelating (Figure 3A,B). Pathway analysis highlighted other pathways where metabolites contribute to the pathway in opposite direction. Citric acid and L‐aspartic acid were mapped to the alanine and aspartate metabolism pathway, and the ratio between the 2 was elevated in both DMD and BMD patients (Figure 3C,D).

**Figure 4:**
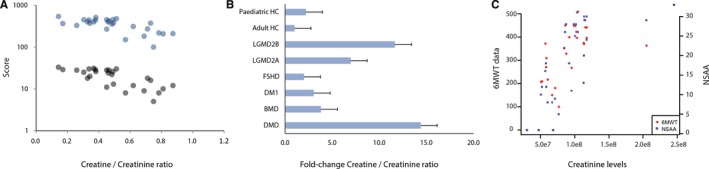
Association of metabolites with clinical data. (A) Scatter plot showing the correlation between the creatine/creatinine ratio and functional scales such as the 6MWT and NSAA. Blue dots represent 6MWT data, while grey dots represent NSAA data. (B) Bar graph showing the fold change in the creatine/creatinine ratio in patients affected by different forms of muscular dystrophies compared to healthy controls. (C) Scatter plot showing the correlation between the creatinine levels and functional scales such as the 6MWT and NSAA. Red dots represent 6MWT data, while blue squares represent NSAA data

The creatine/creatinine ratio has recently been reported to correlate with age in DMD patients suggesting an association with the disease progression.^19^ In our cohort of DMD patients, the time test data including 6MWD and NSAA were available for 27 of 30 patients (3 were non‐ambulant). When we tested the association between the disease severity and the creatine/creatinine ratio, a significant correlation with both 6MWD and NSAA data (*R* = .76 *P *< 10^−6^, *R* = .75 *P *< 10^−5^, respectively) was observed (Figure 4A). The ratio was found to be elevated also in other forms of muscular dystrophy such as LGMD2A and LGMD2B (Figure 4B). When testing for a possible relationship between metabolites levels and clinical scores in DMD patients, only creatinine showed a significant association with both 6MWT and NSAA data (Figure 4C).

## 4 DISCUSSION

DMD is a lethal disease caused by the absence of dystrophin resulting in substitution of muscle mass by adipose tissue.^26^, ^27^ Downstream effects of lack of dystrophin have largely been studied in muscles samples from patients and animal models enabling the identification of morphological alterations and pathological pathways behind the clinical presentation.^28^, ^29^, ^30^, ^31^ Clear metabolic alterations have been described in DMD muscle tissue affecting the energy metabolism (eg glycolysis) and mitochondrial alterations (eg the tricarboxylic acid cycle and electron transport chain). Interestingly, several enzymes involved in these processes have been found to be differentially present in serum and plasma of DMD patients compared to healthy controls, providing evidence that certain metabolic alterations can be detected peripherally by studying protein concentration in serum. Given the very limited data on circulating metabolites concentration in DMD patients, we studied a large proportion of metabolites in fasted patients (to avoid possible food‐related confounders) and compared the signature observed in DMD patients with the profiles observed in the milder allelic form BMD and other forms of muscular dystrophy.

The results of our analysis confirm a decrease in creatinine and an increase in creatine serum levels likely due to the insufficient creatine utilization by muscles.^19^, ^32^ Creatine (from the Greek κρέας, krèas, “meat”) is an intermediate compound of energy metabolism synthesized by the liver (1 g/day) starting from arginine, S‐adenosyl‐methionine and glycine. In mammalian muscles, it serves to regenerate ATP during the first few seconds of muscle contraction. Creatinine, that represents its degradation product and is present in urine and blood, is usually considered a marker of renal function. As in Duchenne patients, creatine is normally synthesized by the liver but not metabolized in muscles, high creatine and low creatinine values are usually observed in both blood and serum. Similar profiles were observed for other forms of muscular dystrophy such as BMD, LGMD2A and LGMD2B. Interestingly, creatinine levels showed intermediate levels in BMD patients compared to DMD and healthy individuals indicating a possible relationship between dystrophin levels in muscle and creatinine in serum. An involvement of the creatine downstream metabolism is supported by the finding that the creatine precursor, the guanidinoacetic acid is reduced in DMD as well as in other MDs. It was recently reported that the creatine/creatinine ratio is particularly elevated in older, more severely affected DMD patients postulating its use as a marker of disease progression.^19^ Our data suggest that there is a negative correlation between the ratio and the performance of patients, as indicated by the significant association with 6MWT and NSAA figures. However, further research in bigger cohorts of patients is needed to confirm this association and its potential use as a surrogate end‐point.

Data regarding the testosterone metabolism confirmed the reduction of 2 testosterone‐related steroids (5α‐DHT and dehydroisoandrosterone 3‐sulphate), most probably caused by treatment with glucocorticoids. We further expand this observation by reporting a reduction in other metabolites involved in testosterone metabolism such as isohomovanillic acid, which is a product of catecholamine metabolism, mainly found in urine as a product of adrenal glands. Reduced levels of isohomovanillic acid could be due to the treatment with glucocorticoids suppressing the hypothalamus‐pituitary‐adrenal (HPA) axis resulting in adrenal glands suppression. More studies are needed to evaluate whether isohomovanillic acid levels could be a prognostic marker of HPA suppression.

Several metabolites involved in amino acids metabolism were affected in DMD patients such as isohomovanillic acid, p‐coumaric acid, L‐Aspartic acid, serine, ornithine, 2‐hydroxycaproic acid and indoleacetic acid. This observation could be the direct effect of increased muscle protein degradation and resynthesis in accordance of what has been observed in dystrophic animal models.^33^

Finally, citrulline levels were found to be reduced in DMD patients; interestingly, citrulline is being tested in a single‐centre, randomized, placebo‐controlled trial in combination with metformin.^34^ This therapeutic approach aims to stimulate mitochondrial function and to compensate oxidative stress by increasing the production of nitric oxide (NO). In fact, NO is synthesized from the precursor arginine, which is in turn synthesized from citrulline.^35^ It was recently shown that both arginine and citrulline can boost the production of NO in humans.^36^ Restoration of citrulline serum levels and NO levels in DMD patients could be used as pharmacodynamics biomarkers to study the effect of this ongoing combination therapy.

While our study is controlled for food intake, other factors such as physical exercise, supplements, stress and time of the day could affect the observed profiles, and they were not investigated in this study. Additional studies in more controlled settings such as clinical trials are required to validate the identified associations.

Despite these limitations our study provides a list of biomarkers useful as potential candidates to evaluate the patients’ disease progression in prospective natural history studies and response to therapy in dose‐finding studies.

## CONFLICT OF INTEREST

The authors report no conflict of interest related to this work.
