# Supplementary figures and images for: Cross‐sectional serum metabolomic study of multiple forms of muscular dystrophy
Source: J Cell Mol Med. 2018 Feb 14;22(4):2442–8. doi: 10.1111/jcmm.13543 (PMC5867073; doi:10.1111/jcmm.13543)

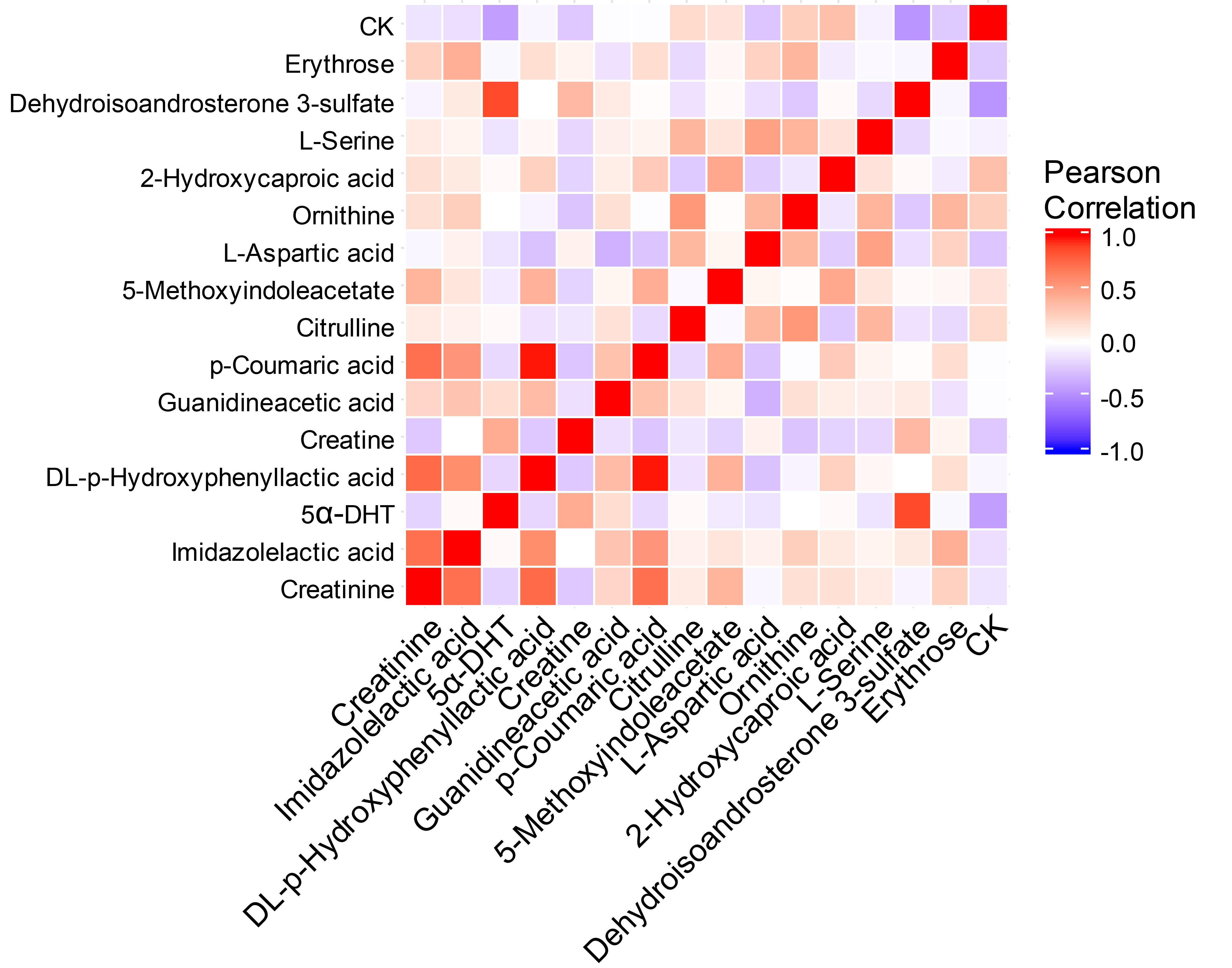

Supplement: Supplementary file 1 [file JCMM-22-2442-s001.tif]

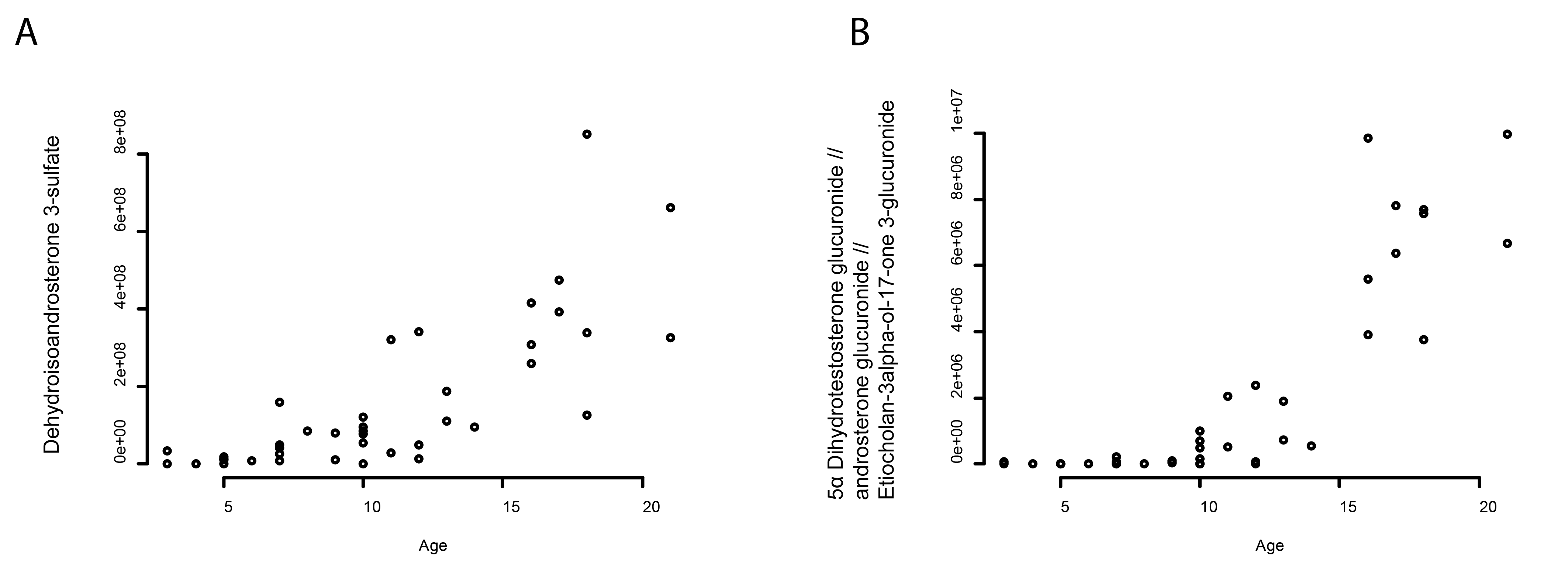

Supplement: Supplementary file 2 [file JCMM-22-2442-s002.tif]
